# Near-Unity Quantum Yields from Chloride Treated CdTe Colloidal Quantum Dots

**DOI:** 10.1002/smll.201402264

**Published:** 2014-10-27

**Authors:** Robert C Page, Daniel Espinobarro-Velazquez, Marina A Leontiadou, Charles Smith, Edward A Lewis, Sarah J Haigh, Chen Li, Hanna Radtke, Atip Pengpad, Federica Bondino, Elena Magnano, Igor Pis, Wendy R Flavell, Paul O'Brien, David J Binks

**Affiliations:** School of Physics and Astronomy and Photon Science Institute, University of ManchesterManchester, M13 9PL, UK; FRS, School of Chemistry, University of ManchesterManchester, M13 9PL, UK; School of Materials, University of ManchesterManchester, M13 9PL, UK; IOM CNR, Laboratorio Nazionale TASC, Area Science Park – BasovizzaS.S. 14 Km. 163, 5 I-34149, Basovizza, (TS), Italy; 5 Department of Chemistry, Vanderbilt University NashvilleTN, 37235 USA and Oak Ridge National Laboratory 1 Bethel Valley Road, Oak Ridge, TN, 37831–6071, USA

**Keywords:** nanocrystalline materials, colloidal quantum dots, photoluminescence, photoelectron spectroscopy, transmission electron microscopy, passivation

## Abstract

Colloidal quantum dots (CQDs) are promising materials for novel light sources and solar energy conversion. However, trap states associated with the CQD surface can produce non-radiative charge recombination that significantly reduces device performance. Here a facile post-synthetic treatment of CdTe CQDs is demonstrated that uses chloride ions to achieve near-complete suppression of surface trapping, resulting in an increase of photoluminescence (PL) quantum yield (QY) from ca. 5% to up to 97.2 ± 2.5%. The effect of the treatment is characterised by absorption and PL spectroscopy, PL decay, scanning transmission electron microscopy, X-ray diffraction and X-ray photoelectron spectroscopy. This process also dramatically improves the air-stability of the CQDs: before treatment the PL is largely quenched after 1 hour of air-exposure, whilst the treated samples showed a PL QY of nearly 50% after more than 12 hours.

## 1. Introduction

Colloidal quantum dots have a number of important optoelectronic applications, including light emitting diodes[1] and photovoltaic (PV) cells.[2–4] Their advantages include cost-effective synthesis, solution processibility and tunable band gaps.[3,4] However, the large surface-area-to-volume ratio of CQDs can result in a high concentration of surface trap states with associated non-radiative recombination pathways. This unwanted recombination reduces PL and electroluminescence QY, charge extraction efficiency and carrier mobility, and thereby limits device efficiency.[5,6]

Trap states are produced by the dangling bonds associated with unsaturated atoms on a CQD surface.[7] Long-chain alkyl ligands have been used to passivate CQDs, i.e. bond with these surface atoms and thus prevent trap formation, but steric hindrance for these bulky molecules results in incomplete coverage of the CQD surface so that some traps remain. Furthermore, long aliphatic ligands act as barriers for charge carrier injection or extraction, inhibiting potential performance in many optoelectronic devices.[8] Charge can be kept away from surface traps by growing a shell of wide-band gap material around the CQD to act as barrier layer. Whilst this can result in high PL QY,[9–11] it also reduces charge extraction and transport which again inhibit device performance.[12] In an effort to reduce surface-trapping without introducing a significant barrier to charge transport, CQDs passivated by short-chain organic ligands have been investigated, and used to sensitize PV cells. However only limited improvements in solar cell efficiencies have so far been achieved using this strategy.[13] Density functional theory has shown that steric hindrance prevents complete passivation even by small molecules, which are unable to penetrate the inter-cation trenches on the surface of the CQDs.[2]

Recently passivation of the surface of some CQD types by chloride ions has been demonstrated to be an effective method of suppressing surface traps without inhibiting charge transport, leading to a record efficiency of 8.55% for a CQD-based solar cell.[14] Chloride ions are sufficiently compact to avoid steric hindrance and to bind to hard-to-reach inter-cation sites.[5] PL QY is a direct measure of surface passivation and to date values of up to 60%[15] have been reported for chloride-passivated CQDs. Chloride treatment not only effectively suppresses surface traps but also increases the stability of the CQDs in air, a benefit of particular importance for the heavier chalcogenides (Se and Te).[16]

Several routes to chloride passivation have been reported using species of varying reactivity. Commonly used examples are molecular chlorine,[16] alkylchlorosilanes[8,17,18] and ionic metal chloride salts.[2,19] The majority of examples of such treatment, to date, have been applied to PbX and CdX (X = S, Se) CQDs. An attempt to passivate PbTe CQDs using molecular chlorine proved unsuccessful as the high reactivity of PbTe towards Cl_2_ led to decomposition and precipitation of the material.[16] Chloride passivation of CdTe CQDs has only once been reported previously.[8] In that case, propyltrichlorosilane, a less reactive reagent than molecular chlorine, was used and resulted in improved photocurrent in CQD films; however, the effect on PL QY was not investigated. We report an alternative and facile chloride treatment of CdTe CQDs using cadmium chloride that results in almost complete passivation, as seen by the observation, for the first time, of a near-unity PL QY in this material. This process also significantly improves stability to air-exposure, increasing the suitability of these CQDs for exploitation in practical devices.[20]

## 2. Results and Discussion

### 2.1. Size, Structure, and Composition

CdTe CQDs were synthesized using a previously reported method.[9] Varying the growth time between 30 s and 6 mins allowed nanocrystals to be grown ranging in diameter from 3.2 nm to 4.9 nm, as determined by the spectral position of the absorption edge (see Supporting Information, SI). The surfaces of these CQDs were treated with chloride ions by injecting a 0.33 mol.dm^−3^ solution of CdCl_2_ in oleylamine containing tetradecylphosphonic acid (TDPA) into a solution of CQDs in toluene at 60 °C, with this temperature held for 15 mins. The quantity of CdCl_2_ injected was increased to allow for the greater surface area of larger CQDs; this calculation is detailed in the SI (see S3).

**Figure 1:**
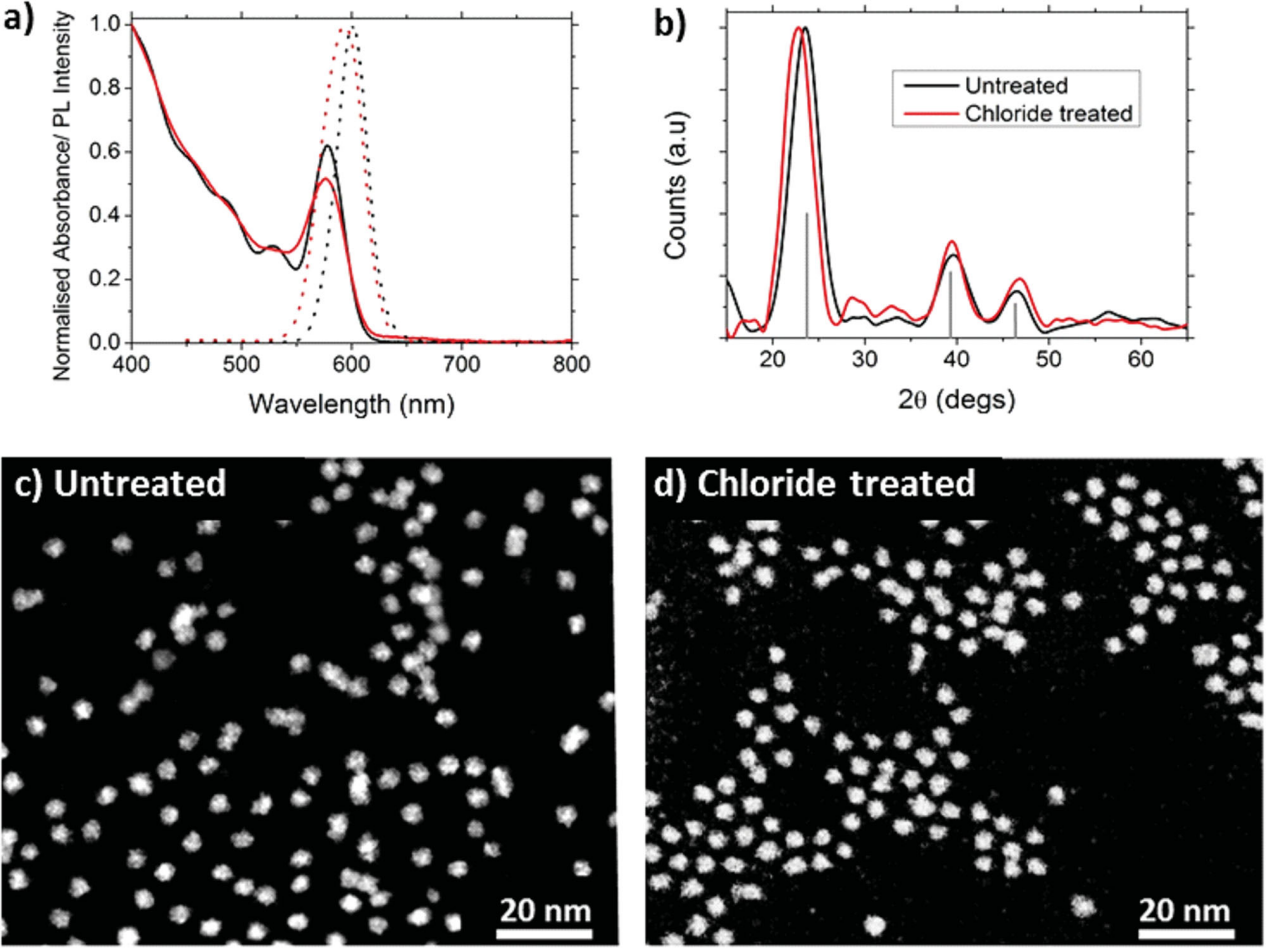
Comparison of CdTe quantum dots before and after chloride treatment. a) absorption spectra (solid lines) and photoluminescence spectra (dotted lines) of both the untreated (black lines) and chloride treated (red lines) CdTe QDs. b) XRD patterns of the untreated (black line) and treated (red line) CdTe samples indexed to zinc blende CdTe. HAADF STEM images of CdTe QDs c) before chloride treatment with an average diameter of 4.6 ± 0.7 nm and d) after treatment with an average diameter 4.5 ± 0.8 nm. In both cases the particles are roughly spherical in shape.

Absorption and emission spectra before and after treatment were taken and showed a small shift in peak position that depended on the reaction time (see Figures S2 a,b in SI) but without any significant change in peak width. For the optimized treatment time and CdCl_2_ concentration used here (see SI), this was typically a shift of a few nm (**Figure** **1**a), indicating that there is little net effect on the band gap, which is ∼2.1 eV for the example shown. The small change in band gap that is observed is likely to be due to the combined effect of several mechanisms, including the change to the CQD size and confinement potential produced by the addition of chloride ions to the CQD surface. The powder X-ray diffraction patterns before and after treatment both agree with that expected for CdTe with a zinc blende structure, confirming that the crystalline nature of the CQDs is unchanged by the treatment (Figure 1b). High angle annular dark field (HAADF) scanning transmission electron microscope (STEM) images show no significant change in size of the CQDs upon treatment (Figures 1 c,d and Supplementary Information S7), consistent with the bonding of chloride ions to the surface atoms of the CQD without the accumulation of a significant CdCl_2_ overlayer. A similar process has previously been shown to result in the effective passivation of PbSe CQDs.[16]

**Figure 2:**
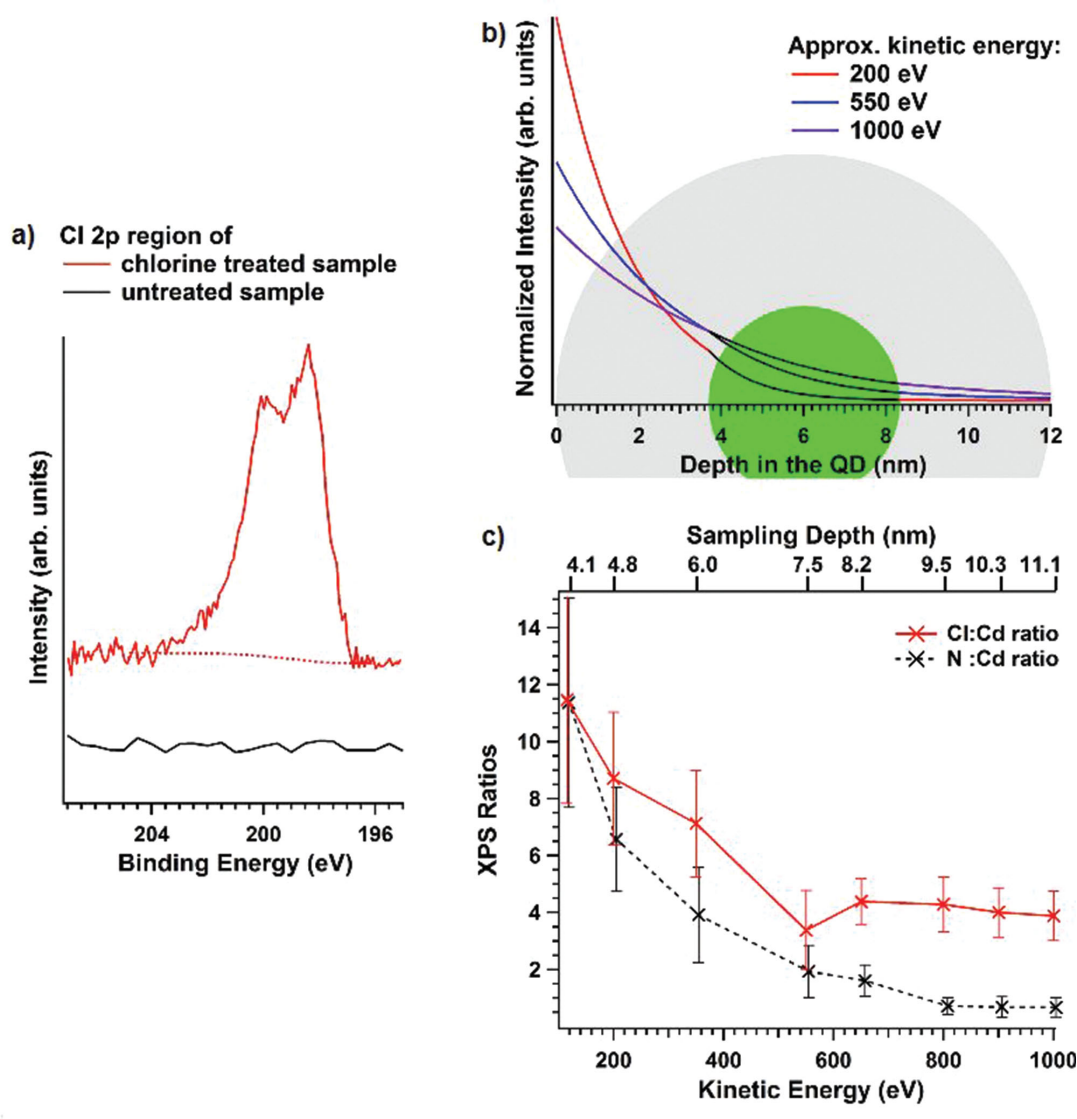
XPS studies showing chlorine present at the surface of the treated samples. a) Cl 2p XPS of CdTe before chloride treatment (black line) and after chloride treatment (red line). b) Schematic diagram illustrating the regions of a spherical CQD (green) surrounded by an organic layer (grey) sampled by photoelectrons at normal emission for different X-ray photon energies. c) Variation of Cl/Cd and N/Cd ratios measured in XPS as a function of photoelectron kinetic energy and hence sampling depth. The data are normalized to the photoelectron flux and the relevant photoionization cross sections; a further experimentally- determined correction has been applied for kinetic energies (KEs) around the Cd MNN Auger energy.

Synchrotron-excited X-ray photoelectron spectroscopy (XPS) was used to confirm the presence of chloride ions on the surface of the treated CQDs. Upon chloride treatment, a Cl 2p doublet peak appears in the spectrum that is not present for the untreated samples (**Figure** **2**a). The depth sensitivity of this feature was investigated by varying the exciting photon energy, which changes the photo­electron inelastic mean free pathlength (and hence the XPS sampling depth) over distances commensurate with the CQD size. Figure 2b indicates schematically the change in sampling depth relative to the diameter of a typical QD, including an outer layer of organic ligands. The thickness of the ligand layer was calculated from the depth dependence of the core and ligand XPS signals, as described in the SI. The variation in intensity of the Cl 2p signal (and the N 1s signal of the oleylamine ligand used in the chloride treatment reaction), relative to the Cd 3d signal, with photo­electron kinetic energy is shown in Figure 2c. It can be seen that the Cl and N signals are strongest (relative to Cd) at the lowest kinetic energy used (115 eV), suggesting that the Cl and N are present largely at the surfaces of the QDs, and strikingly, they are present in roughly equal amounts. This observation is in accordance with previous suggestions that the halide etches the chalcogen at the CQD surface, while the organic ligands remain co-ordinated to the metal.[Bibr b2],[16] The spectra therefore provide confirmation of this model, and suggest that it extends to Cd-containing QDs. Photo­emission was also used to investigate the effect of chloride passivation on the valence band edge, as described in detail in the SI. A significant increase in the number of filled band edge states was observed on chloride treatment, consistent with the passivation of surface traps by donation of electrons from chloride anions. This filling of trap states will not only reduce non-radiative recombination but will also act to narrow the band gap somewhat. This narrowing effect will combine with the influence of change in CQD size and confinement potential on the band gap to produce the net shift in peak PL wavelength observed on chloride treatment.

### 2.2. Photoluminesence Quantum Yield and Decay

**Figure 3:**
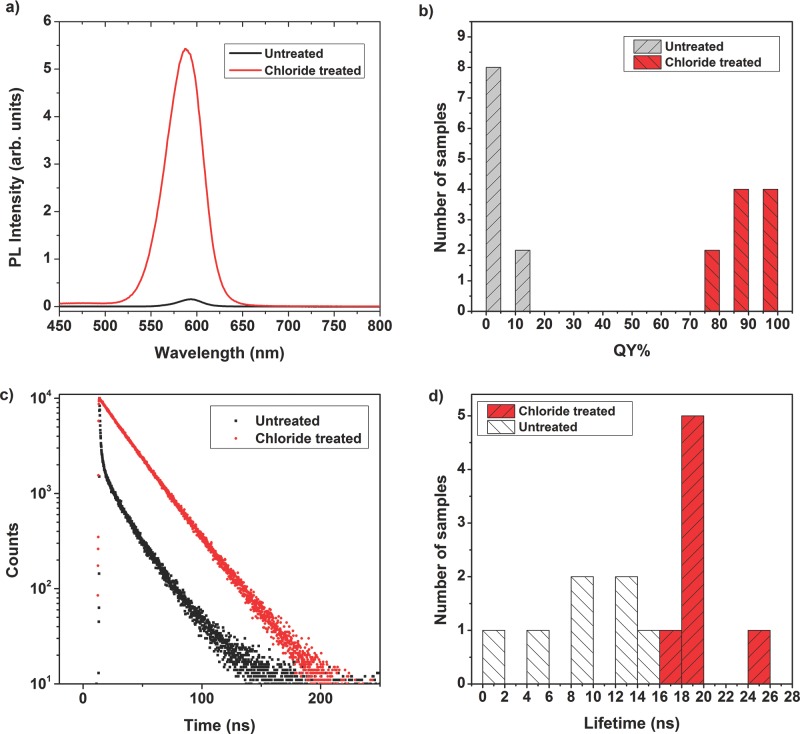
Comparison of QY and PL lifetimes before and after chloride treatment. a) PL spectra for untreated CdTe (black line) and treated CdTe (red line) with the same optical density showing a 20 fold increase in PL intensity upon treatment. b) Histogram showing QYs of ten samples before (grey blocks) and after treatment (red blocks). c) PL decay trace showing multi-exponential decay of a sample before treatment (black points) and the mono-exponential decay of the same sample after treatment (red points). d) Histogram showing the PL lifetimes for samples before treatment (grey blocks) and the increased PL lifetimes after treatment (red blocks).

All samples showed immediate increase in PL intensity upon treatment with CdCl_2_, in some cases by a factor of 20 (**Figure** **3**a). The PL QY for as-synthesized CdTe CQD samples were typically <5%, consistent with similar Cd-based CQDs.[11] These low PL QYs indicate that non-radiative pathways dominate carrier recombination, consistent with the presence of a significant number of surface traps resulting from incomplete surface passivation.

Upon CdCl_2_ treatment these PL QYs rose dramatically, typically to greater than 80%, with the best example reaching 97.2 ± 2.5%, not significantly different from unity (Figure 3b). These results demonstrate that non-radiative recombination is almost entirely suppressed, and that the surface of the CQD can be nearly completely passivated by the CdCl_2_ treatment. Previous examples of near-unity PL QYs of CQDs have been achieved through the growth of a thick inorganic shell to reduce surface defects. This process is potentially advantageous for light emitting diodes (LED) devices,[11] but detrimental in PV cells because the thick inorganic shell acts as a barrier to charge extraction, reducing PV efficiency.[12]

The effect of surface passivation can also be observed using transient PL spectroscopy. The additional recombination pathway afforded by the presence of surface traps not only increases the overall rate of recombination and hence reduces the PL lifetime, but also produces a PL decay transient that is not mono-exponential.[21] Before chloride treatment, the PL transient for all samples was made up of a number of components with differing decay times, including a rapid initial decay component of large relative amplitude, requiring a bi-exponential (or higher order) fit (Figure 3c). After treatment, the PL transient was mono-exponential over three decades of decay (Figure 3c) and the PL lifetime was significantly increased; the PL lifetime of a number of samples before and after treatment are compared in Figure 3d. These results, particularly the mono-exponential form of the PL transient, further demonstrate the effectiveness of this chloride treatment and are consistent with the near-complete passivation of the surface. The increased carrier lifetime resulting from suppression of surface-trap related recombination allows more time for photo-generated charges to be extracted and increases carrier mobility, both of which are likely to improve device performance. Similar significant increases in carrier lifetimes have previously been shown to correlate with performance improvements in inorganic based solar cells.[22]

### 2.3. Effect of Oxygen Exposure

**Figure 4:**
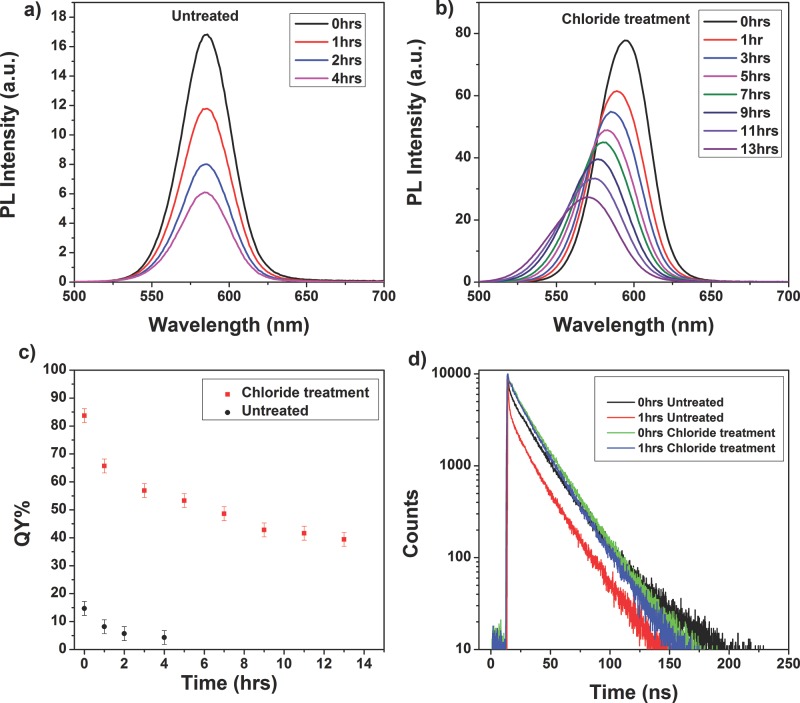
Stability of untreated and treated samples on air-exposure. PL spectra a) before and b) after treatment when exposed to air. c) Decay of QY of untreated (red points) and treated (black points) upon air exposure. d) Transient PL decay traces showing little change upon oxygen exposure for the treated sample (unexposed – green line, 1 hour exposure – blue line) and the formation of rapid non-radiative decay paths upon air exposure for the untreated sample (unexposed – black line, 1 hour exposure – red line).

CdTe CQDs are susceptible to oxidation, with short air-exposure typically leading to nearly complete quenching of the PL.[23] Similarly, we observe a large decrease in PL intensity when the untreated samples were exposed to the atmosphere (**Figure** **4**a). When the chloride-treated samples were exposed in the same way, the rate of decrease in PL intensity was significantly slower (Figure 4b).

For the untreated sample with the highest pre-exposure PL QY (15%), this value decreased to about 5% after 4 hours of exposure (Figure 4c). In contrast, after treatment this sample had a pre-exposure QY of 85% and this was still nearly 50% after 14 hours of exposure. This decay in QY on air-exposure is in contrast to chloride-treated samples kept under inert conditions, which retain their initial QY for many months after synthesis. Interestingly, the PL spectra of the Cl-treated samples also showed a 24 nm blue-shift on exposure to the atmosphere, not seen in the untreated samples, equivalent to a 90 meV increase in band gap. There is a corresponding shift in the absorption edge (Figure S5) which, if it is assumed that the band gap increase is purely due to a reduction in CQD size, can be used to calculate a change in average diameter of 0.16 nm (see SI). However, the band gap could also be affected by the build-up of a wide-band gap oxide layer that increases quantum confinement, and the re-introduction of trap states near to the band edge, as evidenced by the declining QY and is discussed in the SI.

The reduced vulnerability to air-exposure of the treated samples is also evident in the transient PL measurements. The decay transient for the untreated samples showed a significant change after 1 hr air-exposure, with the sharp initial decline accounting for a greater proportion of the total amplitude (Figure 4d), and consequently a reduced PL lifetime and an even greater deviation from the mono-exponential PL decay associated with trap-free recombination. In contrast, after the same time open to the air, the treated sample still exhibits a mono-exponential decay transient with only small increase in the rate of decay (Figure 4d), indicating that far fewer trap sites have been formed by oxygen exposure.

## 3. Conclusions

We have presented a facile CdCl_2_ treatment which can lead to the almost complete passivation of CdTe colloidal quantum dots. This treatment results in near-unity PL QYs being achieved routinely without the formation of a thick inorganic shell. The chloride was shown to reside largely on the surface of the quantum dots, and does not affect crystallinity. The surface passivation of the quantum dots results in non-radiative decay pathways being almost entirely supressed, producing mono-exponential PL decay traces and longer-lived charge carriers as well as ∼100% QY. The suppression of unwanted surface-related recombination promises to lead not only to improved device performance, but will also help further the understanding of charge dynamics in CQDs, the study of which has, up to now, been hindered by the sample-specific contribution of surface processes. The chloride treatment also significantly increased the stability of the CdTe quantum dots when exposed to the air, which will facilitate their inclusion in many optoelectronic devices.

## 4. Experimental Section

All synthetic methods were carried out using standard Schlenk techniques, with anhydrous solvents and an N_2_ atmosphere used throughout the study. Cadmium oxide (CdO, Aldrich, 99.5%), tetradecylphosphonic acid (TDPA, Aldrich, 97%), oleylamine (Acros, 90%), octadecene (ODE, Acros, 90%), trioctylphosphine (TOP, Aldrich, 97%), tellurium (Aldrich, 99.8%) and anhydrous cadmium chloride (Fluka, >99%) were used without further purification.

CdTe CQDs were synthesized using a previously published method[9] using growth times of 30–360 s to produce CdTe CQDs of sizes ranging between 3.2 nm and 4.9 nm, capped with TDPA and TOP ligands. These as-prepared CdTe QDs samples were diluted with dry toluene and stored under N_2_.

A 0.33 M stock CdCl_2_ solution was made by dissolving CdCl_2_ (0.30 g, 1.64 mmol) and TDPA (0.033 g, 0.12 mmol) in oleylamine (5.0 mL). The solution was degassed at 100 °C for 30 min and cooled to 60 °C under N_2_ for the reaction. A toluene solution of CdTe CQDs was held at 60 °C, into which a predetermined amount of CdCl_2_ stock solution, equating to 96 Cl^−^ ions/nm^2^ of CdTe surface area, was quickly injected. The reaction was left for 15 min at 60 °C before being cooled to room temperature and stored as synthesized under N_2_ for further studies.

Absorbance measurements were taken of the samples in a toluene solution using a Thermo Spectronic Heλios β spectrometer. The same solution was used to measure their PL using a Gilden pλotonics fluroSENS fluorimeter after excitation at 400 nm.

X-ray diffraction patterns were measured using a Bruker D8 diffractometer. The samples were prepared by depositing a concentrated hexane solution on glass slides and allowing the sample to dry in air. This was repeated until a thick film was deposited suitable for X-ray measurements.

Samples were prepared for scanning transmission electron microscope (STEM) analysis by drop-casting a suspension of the CQDs onto a holey carbon support film which was then washed with several drops of methanol. High resolution TEM (HRTEM), high angle annular dark field (HAADF) STEM images, and energy dispersive X-ray (EDX) spectrum images were acquired using a probe side aberration corrected FEI Titan G2 80–200 kV operated at 200 kV with a convergence angle of 26 mrad and a HAADF inner angle of 52 mrad. Further HAADF STEM imaging and electron energy loss spectroscopy (EELS) was performed using an aberration corrected NION UltraSTEM200 equipped with a Gatan Enfinium EEL spectrometer and operated at 200 kV. Cd M_45_, and Te M_45_ edges were used for the EELS elemental maps. Before imaging in the UltrasSTEM200, the samples were baked in vacuum at 160 °C for 8 hours to remove surface ligands. Particle size analysis was performed by applying a threshold to the HAADF STEM images using ImageJ software. Over 200 particles from each sample were measured.

For XPS measurements, CQD samples were examined both with and without the Cl passivation treatment. In the case of the latter, the initial TOP and oleylamine ligands were ligand-exchanged for *n*-butylamine, in order to avoid charging in XPS.[24] The CQD samples were deposited from solution in methanol onto ITO (tin-doped indium oxide) glass slides, and mounted on the sample holders for UHV (ultrahigh vacuum) experiments using carbon tape and silver-loaded UHV-compatible paint. XPS was performed using the BACH beamline (35<hυ<1600 eV), which is equipped with a 135 mm hemispherical electron energy analyzer (Scienta R3000, VG Scienta), at the Elettra synchrotron in Trieste, Italy. XPS spectra were recorded at room temperature at a total instrumental resolution of 201 meV (at 320 eV photon energy) to 1.42 eV (at 1490 eV photon energy). Data were fitted with product (70% Gaussian/30% Lorentzian) curves and a Shirley-type background was subtracted, except for the Cl 2p region at 200 eV KE where a linear background was chosen. Binding energies of spectra were calibrated to literature values for the CdTe Cd 3d doublet. Spin-orbit separations for the Cd 3d, Te 3d and Cl 2p doublets were set as 6.75 eV,[25] 10.38 eV[25] and 1.6 eV[26] respectively, and the chemical shift between the two Cl 2p components was fixed to 1.22 eV.

PL transients for CQD samples in toluene were recorded using the time correlated single photon counting technique. A mode-locked Ti:sapphire laser (Mai Tai HP, Spectra-Physics) was used to produce ∼100 fs pulses at 80 MHz repetition rate and a wavelength of 820 nm. This rate was reduced to 2 MHz by an acousto-optic pulse picker (Pulse Select, APE) before the wavelength was converted to 410 nm via second harmonic generation (APE Harmonic Generator). These pulses were used to excite the sample contained in a quartz cuvette, sealed under nitrogen. The resultant emission was passed through a monochromator (Spex 1870c) tuned to the PL peak before detection by a multi-channel plate (Hamamatsu R3809U-50). The time correlation of the detected photons was performed using a TCC900 PC card from Edinburgh Instruments.

PL QY for the CQD samples in toluene was measured using a spectrofluorometer (FluoroLog 33–22iHR, Jobin-Yvon) with a built-in integrating sphere (F-3018, Jobin-Yvon). The excitation wavelength was set to 450 nm with a bandwidth of 1.3 nm. To verify the procedures used, the QY was also measured for 4 different dye standards emitting at similar wavelengths and similar efficiency to the QDs under study. The QY values for Rhodamine 6G (Lambda Physik), Oxazine 1 (Lambda Physik), Pyromethene 580 (Exciton Inc) and Pyrromethene 605 (Exciton Inc.) were measured to be 97.6%, 13.0%, 90.1% and 72.9%, respectively. The standard error calculated for 5 repeats of these measurements was 2.5% and so these results agree well with the literature values i.e 95%,[27] 14.1%,[28] 90%[29] and 74%,[29] respectively.
