# Supplementary material for: Near-Unity Quantum Yields from Chloride Treated CdTe Colloidal Quantum Dots
Source: Small. 2014 Oct 27;11(13):1548–54. doi: 10.1002/smll.201402264 (PMC4409856; doi:10.1002/smll.201402264)
Supplement: Supplementary file 1 [file smll0011-1548-sd1.pdf]

Copyright WILEY-VCH Verlag GmbH & Co. KGaA, 69469 Weinheim, Germany, 2014.

NANO small MICRO

Supporting Information

for *Small.,* DOI: 10.1002/smll.201402264

Near-Unity Quantum Yields from Chloride Treated CdTe Colloidal Quantum Dots

*Robert C. Page, Daniel Espinobarro-Velazquez, Marina A. Leontiadou, Charles Smith, Edward A. Lewis, Sarah J. Haigh, Chen Li, Hanna Radtke, Atip Pengpad, Federica Bondino, Elena Magnano, Igor Pis, Wendy. R. Flavell, Paul O'Brien, and David J. Binks**

Supporting Information

S1. Optimizing concentration of $CdCl_2$ used

Five separate quantities of $CdCl_2$ were injected into a 70 µmol solution of 3.5 nm diameter CdTe colloidal quantum dots (CQDs), corresponding to a range of 24-120 Cl^- per nm^2 of CQD surface area (0.1-0.5 mL $CdCl_2$ solution per mL of CdTe CQD solution). The addition of the smallest amount of $CdCl_2$ used, corresponding to 24 Cl^- per nm^2, resulted in an immediate apparent increase in photoluminescence (PL) from the CQDs. This was confirmed by measuring PL quantum yield (QY) which rose to over 80% from <2%. The PL QY increased upon adding more $CdCl_2$ until reaching a maximum of 97% for 96 Cl^- per nm^2, decreasing again on the addition of further $CdCl_2$ – see **Figure S**1.

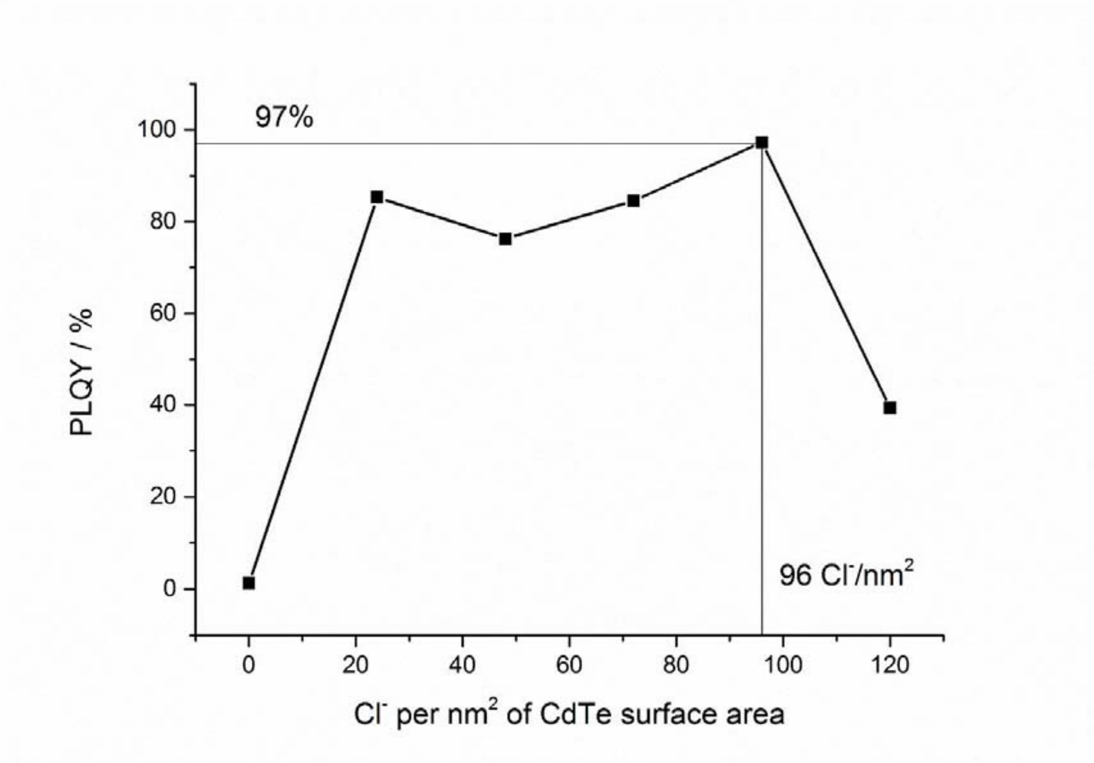

Figure S1. PL QYs for different concentrations of $CdCl_2$ solution added.

S2. Optimizing reaction time

A sample of 3.5 nm diameter CdTe CQDs was treated by the injection of $CdCl_2$/TDPA/oleylamine solution at 60°C at a concentration corresponding to 96 Cl^- per nm^2, i.e. the optimum value as determined above. Aliquots of the reaction solution were extracted throughout the reaction at various times spanning a total of 4 hrs so that the absorption and luminescence spectra could be monitored – see **Figure S**2. After 1 minute of reaction time there is an immediate increase in brightness evident to the eye, with PL intensity (for the same concentration of CQDs) increasing eight fold when compared with the untreated sample. This increase in brightness coincides with a small blue-shift in the PL peak of 8 nm. A similar blue-shift has been observed previously in similar CQD systems and attributed to a surface etching of the CQD reducing the optically active size of the CQDs.[1,2] After this, there is a monotonous red-shift of the PL peak with reaction time, first back to the position of the untreated sample and then, after 4 hrs, 6 nm beyond it. This is attributed to an increasingly thick $CdCl_2$ shell being grown. The absorbance spectrum followed a similar pattern of spectral shifts. **Figure S**1c show the ratio of maximum PL intensity and the absorbance at the excitation wavelength of 400 nm. This indicates that the QY reaches a maximum after 15 mins reaction time before decreasing somewhat and then rising again and then remaining approximately constant. Since a thick layer of $CdCl_2$ is likely to inhibit charge transfer into and from a CQD and the monotonous red-shift of the PL and absorbance spectra with reaction time, after the initial blue-shift in the first minute, indicates that a $CdCl_2$ layer is continuing to grow, 15 min was chosen as the optimum reaction time.

Thus, for all other treatments used in this study the reaction was carried out at 60 °C for 15 mins using a chloride precursor concentration corresponding to 96 Cl^- per nm^2 of CdTe CQD surface area.

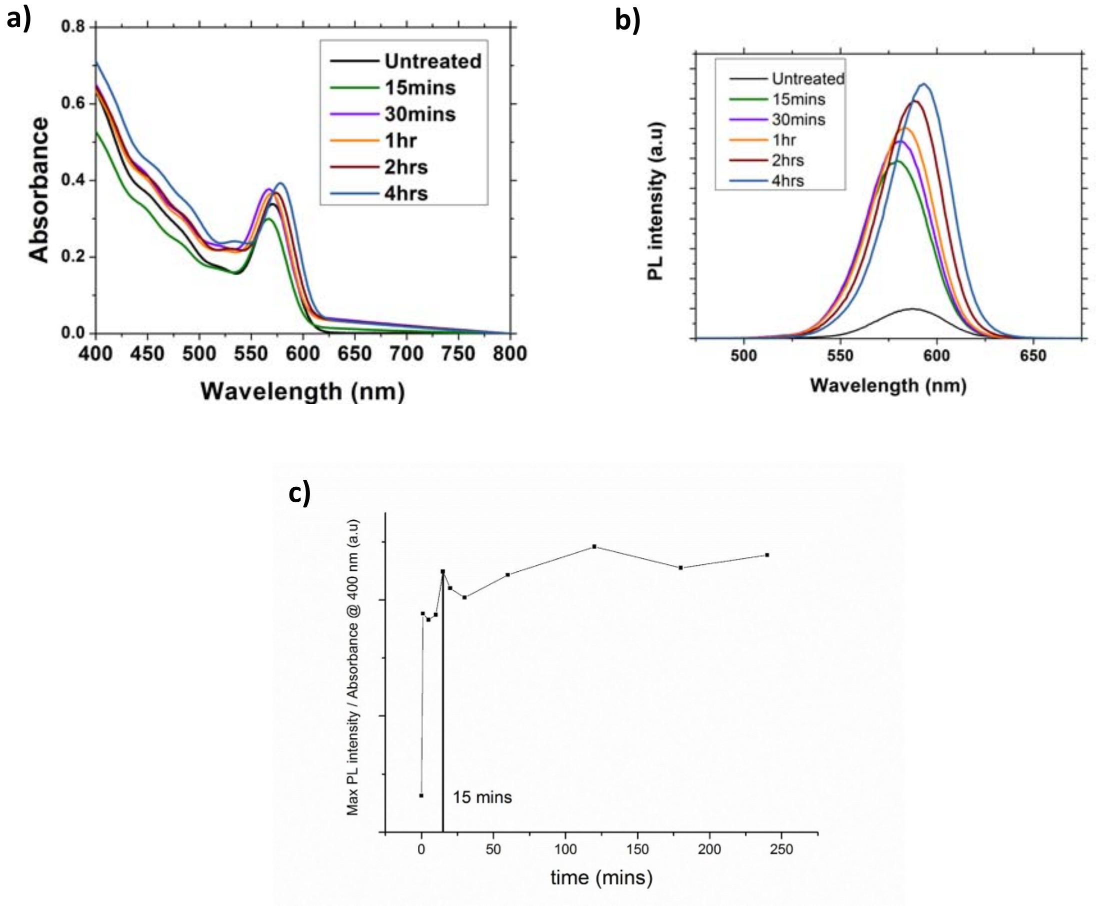

Figure S2. Optimizing $CdCl_2$ reaction time. a, Absorption and **b**, photoluminescence spectra with respect to chloride treatment time. **c**, Ratio of photoluminescence intensity, to absorbance at 400 nm. (Some spectra have been omitted from **a** and **b** for clarity).

S3. Calculating the amount of $CdCl_2$ solution to add

The size of the CdTe CQDs was calculated using the following formula[3]

$$D = (9.8127 \times 10^{-7})\,\lambda^3 - (1.7147 \times 10^{-3})\,\lambda^2 + 1.0064\,\lambda - 194.84 \qquad (1)$$

where D is the average diameter of the CdTe CQDs (in nm) and λ is the wavelength (in nm) corresponding to the absorption band edge.

The concentration of a CdTe CQD solution, C, (in units of $mol.dm^{-3}$) was calculated by [1]

$$C = \quad A/(10043\times D^{2.12})L \qquad (2)$$

where A is the absorbance at the absorption band edge, and L is the path length through the solution.

The total surface area of CdTe CQDs in solution, SA, is calculated from the product of the surface area of a single CQD and the concentration and volume, V, of the solution:

$$SA = 4\pi\,(D/2)^2 \times C \times V \qquad (3).$$

Hence, the volume of 0.33 mold.m^{-3} $CdCl_2$ solution used in a chloride treatment, V_{Cl}, so that it corresponds to 96 Cl^- per nm^2 of CQD surface is calculated to be

$$V_{Cl} = 48 \times SA/0.33 \qquad (4).$$

S4. Analysis of X-ray photoelectron spectroscopy data.

The sampling depth in X-ray photoelectron spectroscopy (XPS) is defined as 3 times the inelastic mean free path, 3λ; 95 % of the signal originates from this region. Given that the photoelectron flux emerging from the sample drops exponentially with distance below the surface, all the measurements described here primarily probe the first few nm of the nanoparticle surface, as 63% of the signal is received from within a distance λ of the surface. In a typical experiment, electron kinetic energies between 115 eV and 1000 eV were probed, corresponding to sampling depths of 4.1 – 11.1 nm. This enabled us to quantify the surface composition of the QDs.

Figure S3 shows the XPS data used to obtain the composition *vs.* depth plot in **Figure 2**c: the Cd 3d, N 1s and Cl 2p spectra for a sample of 4.5 nm diameter Cl-passivated CdTe CQDs as a function of photoelectron kinetic energy, and hence sampling depth. It can be seen that the Cl 2p and N 1s signals are strongest (relative to Cd) at the lowest sampling depth used (4.1 nm), suggesting both are localized at the surface of the QDs. Two Cl 2p signals are observed; of these, the lower binding energy (BE) component with Cl $2p_{3/2}$ at *ca.* 198 eV has a BE consistent with $CdCl_2$. Both signals increase relative to Cd 3d at low sampling depth, so we associated the higher BE component with another form of surface-adsorbed Cl. The N 1s peak contains three components; a broad feature centered at *ca.* 397 eV BE contains peaks due to N in the oleylamine ligand and the protonated ligand at higher BE, while a further small component is present at *ca.* 402 eV BE. The latter does not

change in intensity relative to Cd with sampling depth, and is consistent with a second CdN phase; it is omitted from the analysis in **Figure 2**b. Small depth-dependent signals due to P 2s were also detected, and are associated with residual TOP ligand and TDPA.

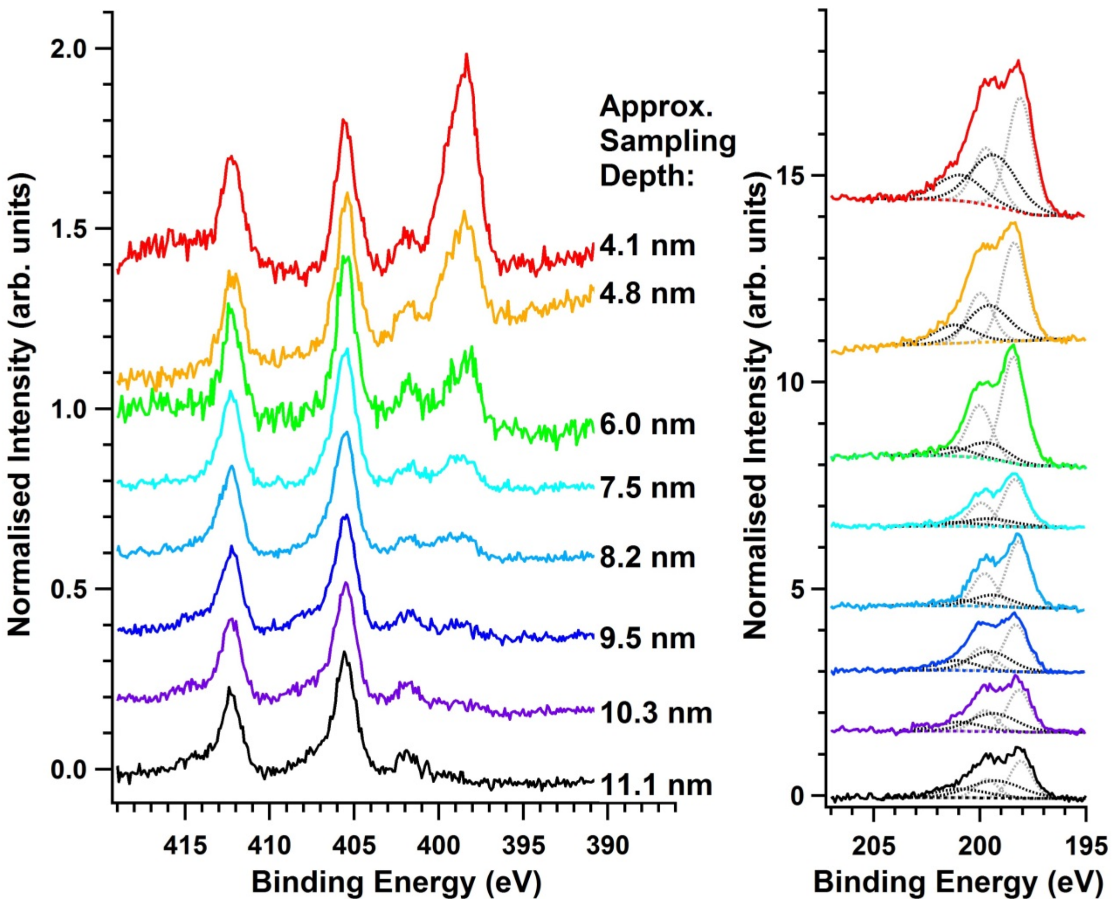

Figure S3. a, Cd 3d (*ca.* 405, 412 eV binding energy) and N 1s (centered at *ca.* 400 eV binding energy) and, **b**,Cl 2p XPS spectra for 3.5 nm Cl-passivated CdTe QDs as a function of photoelectron kinetic energy (from 115 eV (red) to 1000 eV (black)) and hence sampling depth. The sample has been exposed to air after deposition for 20 min. The spectra are normalized to the Cd $3d_{5/2}$ intensity at *ca.* 405 eV binding energy, and are corrected for photoionization cross section, except that of N 1s.

As the flux of photoelectrons is attenuated according to the Beer-Lambert law as it emerges from the sample, it is possible to obtain an estimate of the thickness of the ligand shell by a simple model incorporating the inelastic mean free paths. We adopt a 'two-layer model' that assumes a ligand shell overlies the core, and use the analytical model developed by Shard *et al.* which accounts for a surface made up of spherical particles with radii of the same order as the inelastic mean free

path length[4-6]. This calculation requires us to estimate the number of organic ligands (oleylamine, TOP and TDPA) coordinated to the surface of the QD. Using the relative intensities of the C 1s, N 1s, P 2s, Cl 2p and Cd 3d signals as a guide, we carried out ligand shell thickness calculations for a number of reasonable Cd:C ratios (from 1:1 to lower coverages corresponding to 2:1, by analogy with previous work[7]). For each, we calculated the ligand shell thickness for 6-8 different sampling depths; in principle the thickness obtained should be independent of the sampling depth used (within experimental error), and this test gives a useful indicator of the validity of the model chosen. In order to obtain this condition, we found it was necessary to assume a Cd:C ratio of *ca.* 1:1, i.e. roughly one organic ligand is bound to each surface CdTe unit. We obtain a ligand layer thickness of *ca.* 3.7 nm for a CdTe QD diameter of 4.5 nm. When this is compared with the length of the longest ligand (TDPA, *ca.* 2.1 nm), it can be seen that the calculated average organic layer thickness is more than one ligand layer. We assume that this corresponds to one layer of directly coordinated ligands with some excess ligand interdigitated into the ligand shell (consistent with the observation of signals from both protonated and unprotonated oleylamine), together with residual solvent molecules.

S5 Valence band photoemission studies.

In order to study the bonding of the chlorine at the CdTe nanoparticle surfaces, we have carried out valence band photoemission studies (**Figure S4**). Unpassivated CdTe QDs react swiftly to form surface oxide in air, even during the short (few-minute) period of air exposure necessary to insert the samples into the UHV spectrometer (as evidenced by a strong Te 3d doublet lying 3.6 eV to higher binding energy than the CdTe signal in core level XPS, assigned to TeO_2 [9]). For this reason, we have carried out our examination of the effect of chloride anion passivation using CdTe and various more stable chalcogenide QD systems. These include CdTe/CdSe and CdTe/CdSe/CdS core-shell structures, where outer stabilising CdSe or CdSe/CdS shells are grown before Cl anion

passivation. Representative examples are shown in **Figure S4**. In the case of shelled systems, such as CdTe/CdSe/CdS, we observe a valence band onset at around 1 eV BE, consistent with the VBM expected for this system. After chloride anion passivation of CdTe/CdSe, significant new filled states are observed at the valence band edge, tailing to the Fermi level. The edge position is not significantly changed when the sample is aged in air for 66 hours. In the case of aged CdTe, the valence band edge again shows an onset at around 1 eV BE, which rises to higher binding energy. XPS shows significant TeO_2 peaks (which has an optical band gap of around 4 eV [10]), so the states above 4 eV BE at least in part reflect the valence band of the oxide. Again, on Cl anion passivation, significant new states appear at the valence band edge, consistent with the results from the shelled systems. The observation of these new states in the band gap suggests band gap narrowing (i.e. a red shift) occurs during the Cl-passivation process, and this is consistent with our experimental observations (see **Figure 4** in themain text). The substantial increase in the valence band density of states at around 4 eV BE is consistent with DFT calculations for Cl-passivated PbS. [11] In the unpassivated QDs, surface states are thought to give rise to reactive partially-filled states in the band gap, which act as trapping sites.[7] In the chloride anion passivation process, we expect the Cl^- ion to donate electrons into these states, giving rise to *new filled states* (removing some empty states) in the band gap. These filled states are directly probed by the photoemission process. We note that DFT calculations for chloride-passivated Si nanocrystals also predict the observed band gap narrowing on Cl-passivation[12] We therefore propose that Cl bonds to the surface by the anion donating electrons into partially-filled reactive surface states, creating new completely filled states. Although the noise level is significant, it appears that on ageing the CdTe/Cl, there may be a small decrease in the density of states at the VBM (at around 1-2 eV BE), which is consistent with the small blue shift observed over time in the PL of aged samples (see **Figure 4** in the main text, and **Figure S5** below). This is consistent with the onset of some initial oxidation resulting in a small decrease in size of the CdTe 'core' of the QDs as its surfaces become oxidized. This blue shift due to oxidation has been observed in a number of other chalcogenide systems. [13,14]

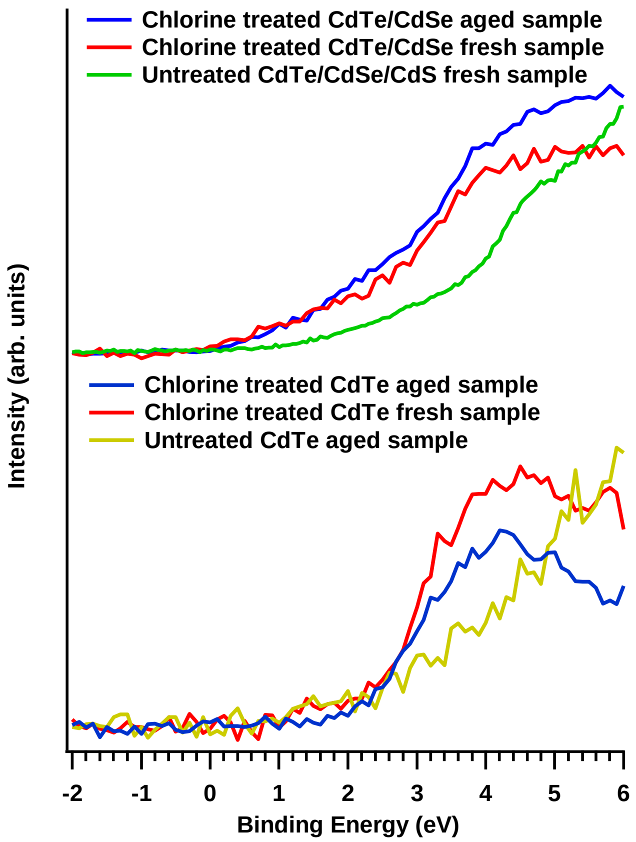

Figure S4: Valence band photoemission spectra recorded at 370 eV (top panel) and 850 eV (bottom panel) near the valence band maximum for unpassivated and chlorine-passivated chalcogenide QDs. The 'aged' samples have been exposed to air for *ca.* 66 h, while the fresh samples are exposed to air for 15 - 90 min during sample mounting and pump-down of the spectrometer load-lock. The data are normalized to the Cd 4d intensity in all cases, and referenced to the Cd 4d BE. [8]

S6. Effect of air-exposure on the absorption spectrum.

Figure S5 shows the change in absorbance spectra on oxidation for the same sample used to produce **Figure 4**b in the main manuscript. The position of the first absorption peak decreases from 582 nm to 562 nm over 13 hours of air-exposure. If it is assumed that this shift in wavelength is caused by a change in CQD size then, using equation (1) above, this corresponds to a reduction in diameter of 0.16 nm.

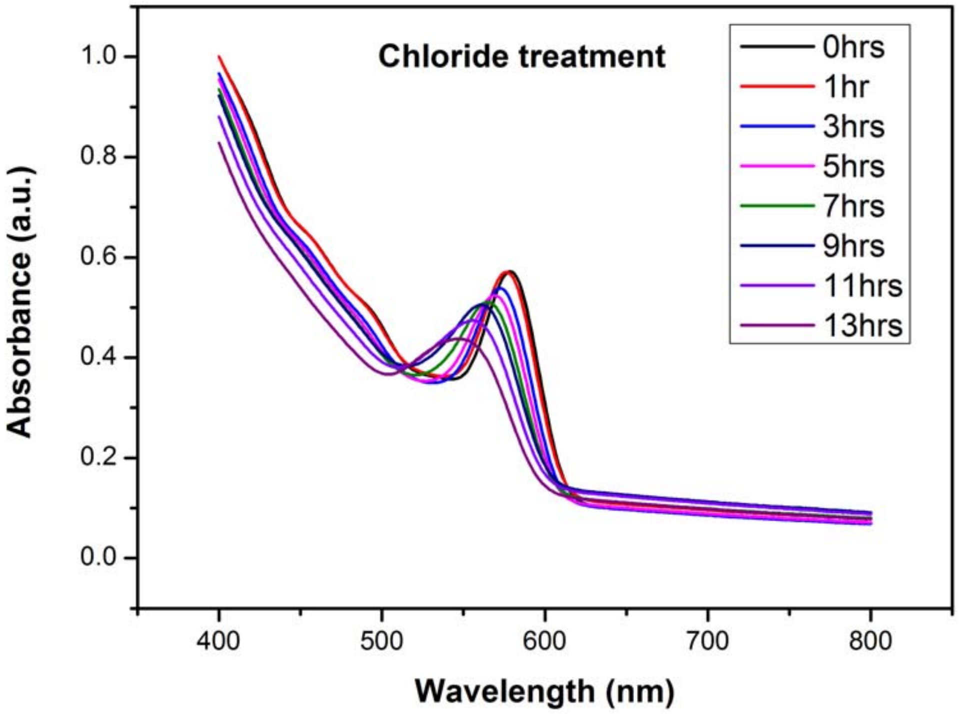

Figure S5: The change in the absorbance spectrum of a chloride-treated sample on air-exposure.

S8 High resolution (scanning) transmission electron microscopy

High resolution electron microscopy was used to probe the crystallinity and chemistry of the nanocrystals at the individual particle level. Fig. S6 shows high resolution transmission electron microscope (HRTEM) images of typical particles from the treated CdTe sample and the corresponding Fourier transforms which have been indexed to determine the crystal structure and orientation of the particles. The Scanning transmission electron microscope (STEM) was also used in order to acquire high resolution high angle annular dark field (HAADF) images (Fig. S7) and tp perform electron energy loss spectroscopy (EELS) elemental mapping of individual nanoparticles (Fig. S8).

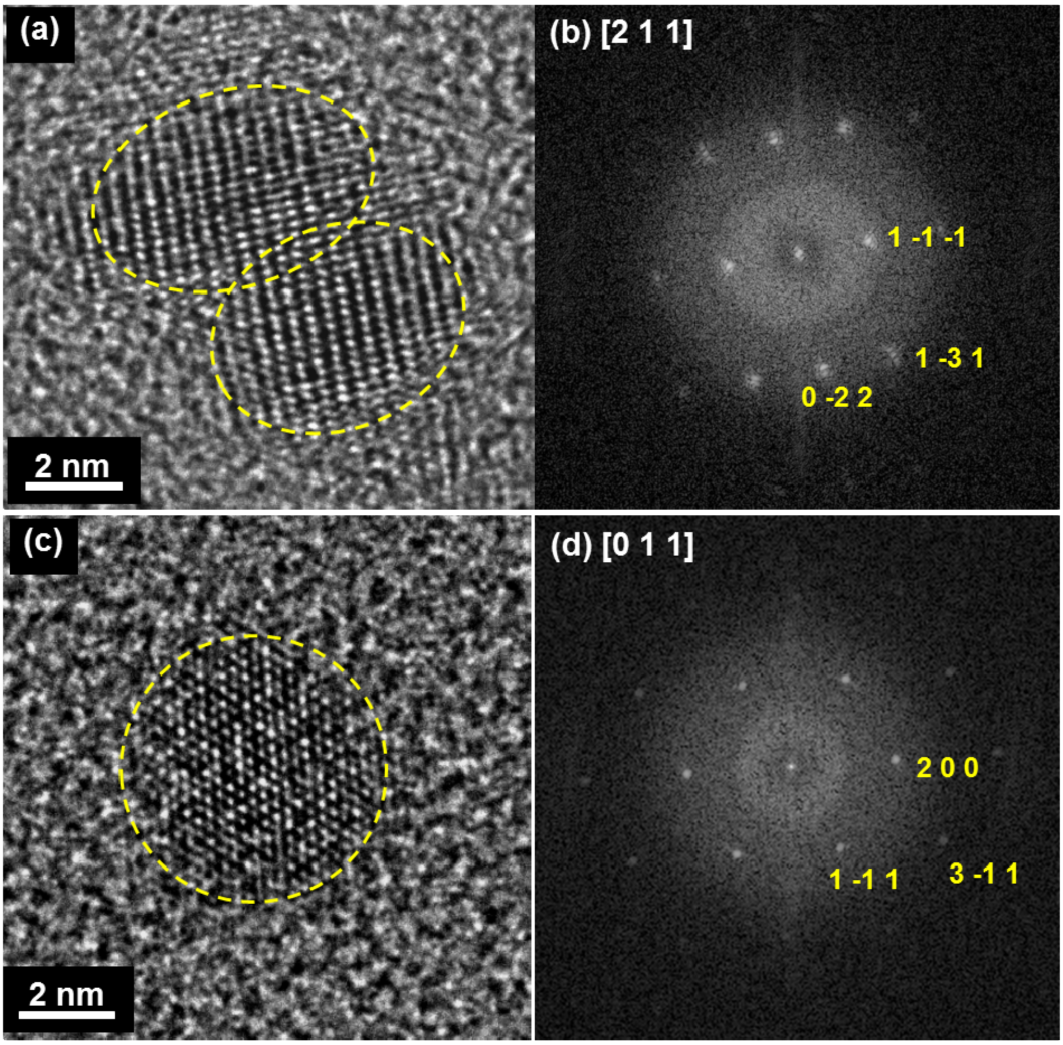

Fig S6 HRTEM images. High resolution TEM (HRTEM) images of the treated CdTe nanoparticle sample. (a) Shows a pair of agglomerated nanoparticles with the Fourier transform shown in (b) which has been indexed to the CdTe Zinc Blende structure viewed along [2 1 1]. (c) Shows a nanoparticle in the same sample which has oxidised (the Fourier transform shown in (c) has been indexed to the CdO structure viewed along [0 1 1]).

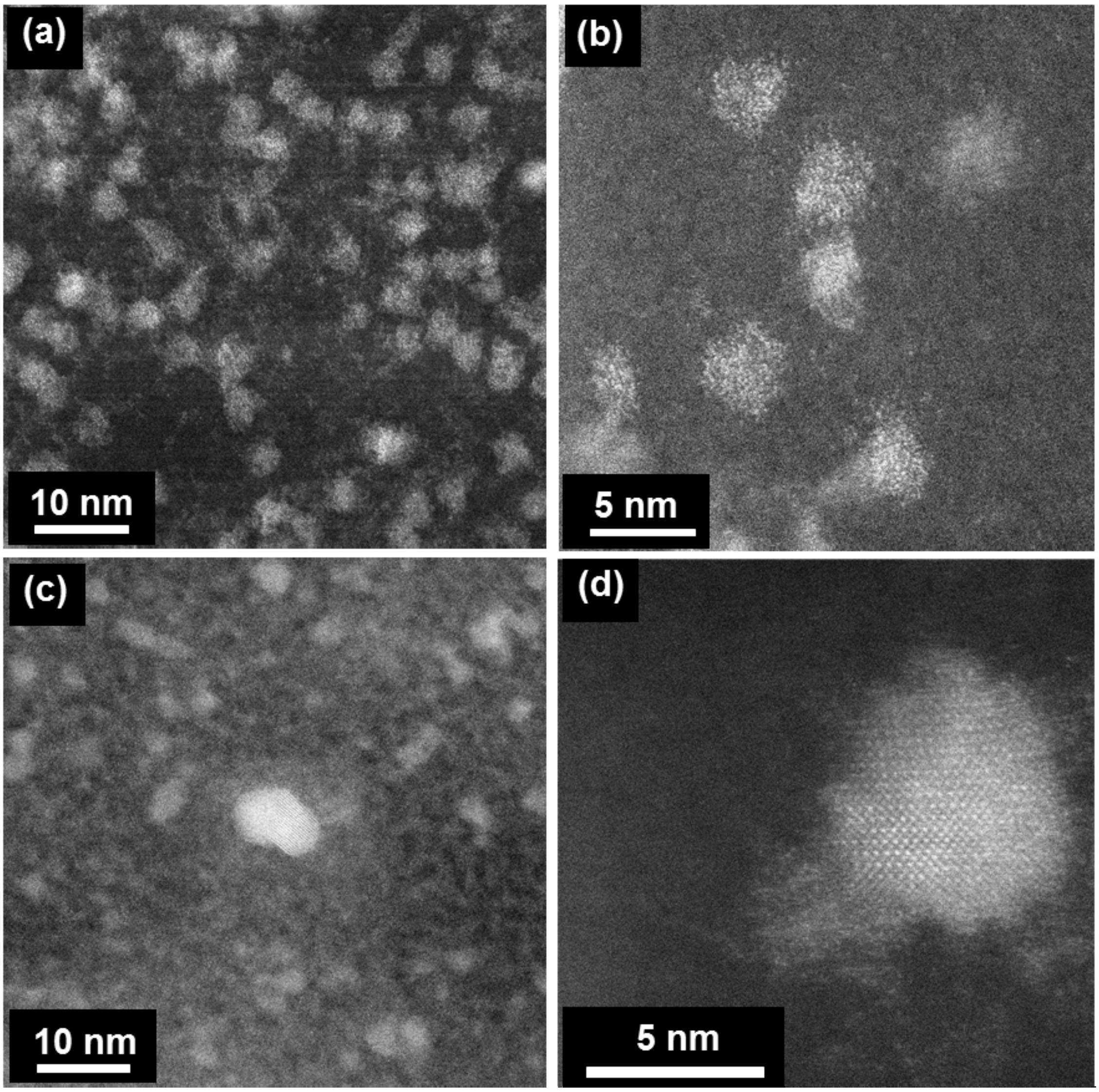

Fig S7 High resolution HAADF STEM images. High resolution STEM images of the CdTe untreated (a,b) and treated (c,d) nanoparticles after baking in vacuum at 160°C for 8 hours to remove surface ligands. Many of the particles are amorphous with some larger nanoparticles likely to be CdO.

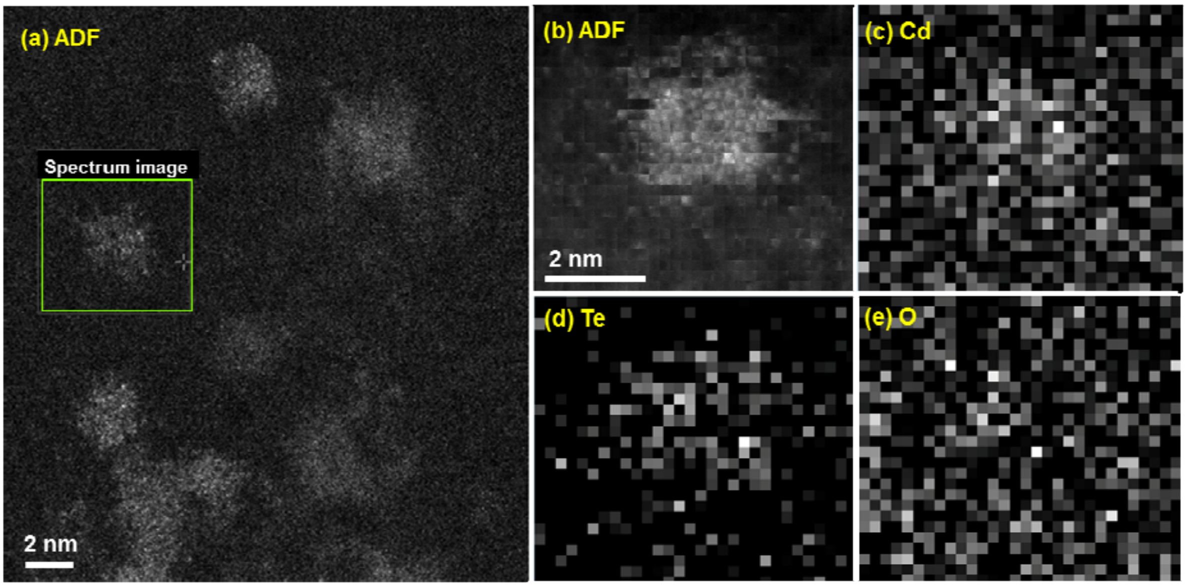

Fig S8 Electron energy loss specroscopy (EELS) elemental mapping. High resolution STEM imaging (a,b) and EELS elemental mapping (c,d,e) for an untreated CdTe nanoparticle after baking in vacuum at 160°C for 8 hours. The area of the EELS spectrum image is shown by the green square in (a). As expected the particle contains cadmium and tellurium while oxygen (and carbon) are not localised within the nanoparticle but associated with the carbon support film and remaining surface ligands. Elemental maps were extracted after appropriate background subtraction for energy windows of (a) 444-528eV, (b) 588 – 678eV and (c) 533-563 eV. This elemental analysis is in agreement with energy dispersive x-ray (EDX) spectroscopy data for the untreated sample which showed only cadmium and tellurium signals associated with the nanoparticles. All other elemental signals present in the EDX spectra (oxygen, carbon and copper) are known to originate either from the surface ligands or from the holey carbon/copper TEM support grid.

S7 Particle size analysis

In order to gain an accurate picture of the size and shape of the nanoparticles automated image analysis was performed on a large number of HAADF STEM images. The HAADF STEM imaging mode was chosen in preference to conventional bright field TEM images as these images give considerably higher contrast between the nanoparticles and the carbon support. Automated image analysis allows several hundred particles from each sample to be measured in a consistent manner, generating statistically significant data about the nanoparticle size and shape distributions. Fig. S9 shows the automated image processing procedure and Fig. S7 shows the resulting data, represented in histograms showing the spread of particle sizes and aspect ratios in both samples.

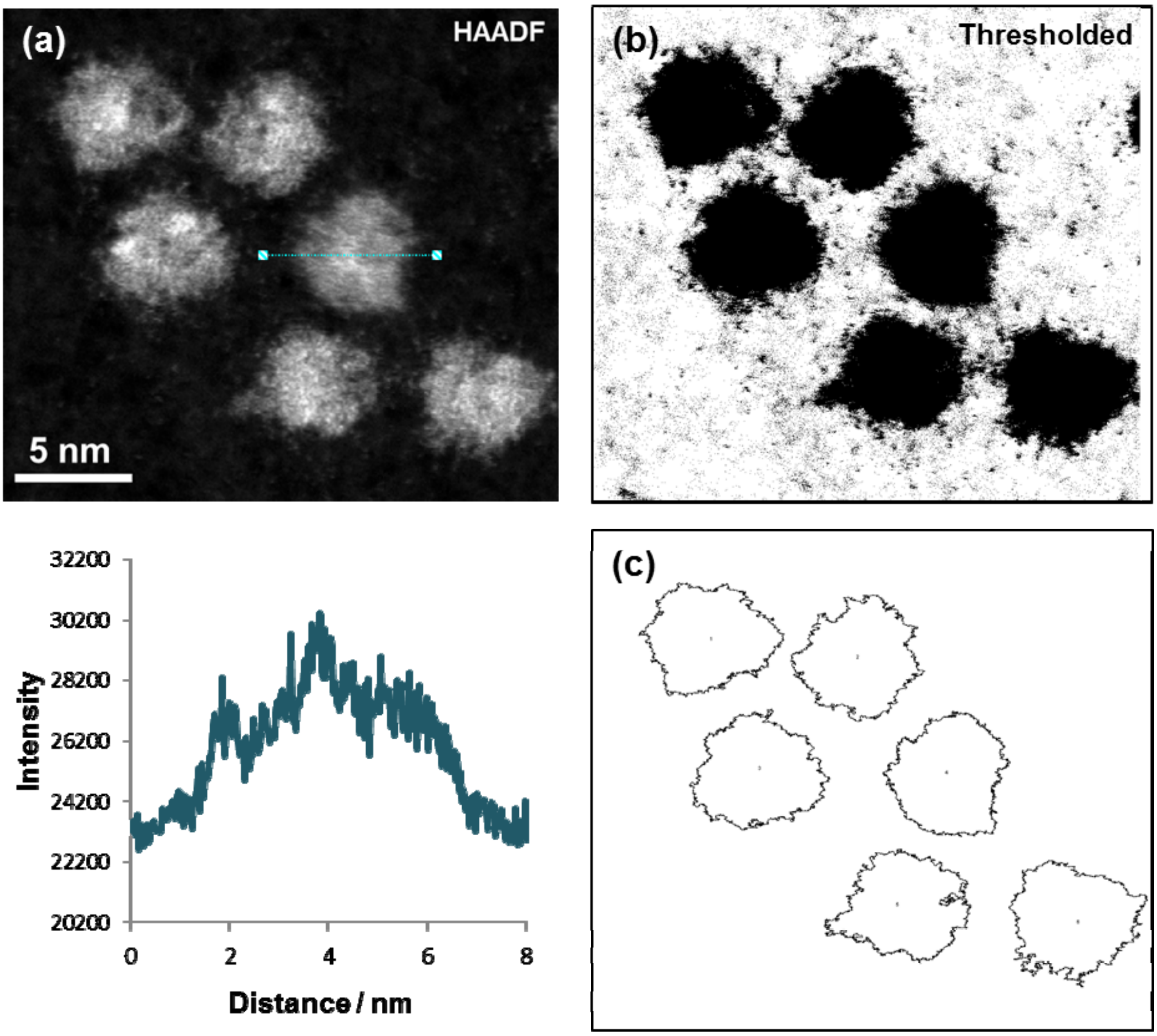

Fig. S9 Automated size analysis of HAADF STEM images. As the line scan in (a) demonstrates, the intensity change going from the support to the particle is not a sharp step profile but a continuous gradient, this is due the spherical particles being thinnest at their edge and thickest in the centre. Defining the edge of a particle for size analysis is therefore not entirely straightforward and a consistent definition of the edge is required for automated image analysis. Automated size analysis was performed in the ImageJ software after the HAADF STEM Images were converted to 8-bit tiffs. The mean intensity for the carbon support was identified and subtracted as a constant background. The threshold intensity value could then be set as 10% of the maximum intensity to create thresholded images like that shown in (b). ImageJ particle analysis was then applied (c) with maximum and minimum areas defined as 30 nm^2 4 nm^2 respectively so as to avoid the detection of single heavy atoms as small particles or touching neighbours as large particles. This approach allowed the rapid analysis of several hundred particles from each sample.

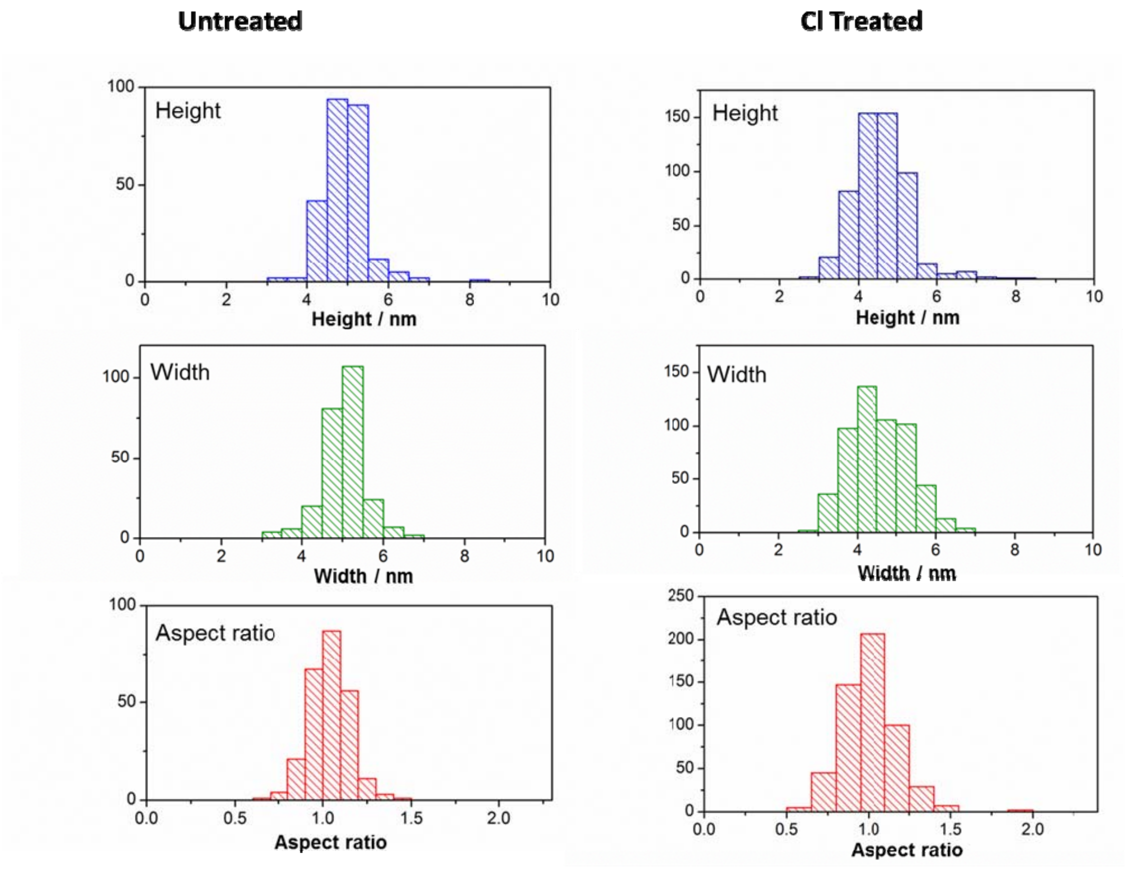

Fig S10 Analysis of the size and morphology of nanoparticles from HAADF STEM images. Based on the automated size analysis procedure outlined in Fig. S6, we were able to perform size analysis on 251 particles from the untreated sample (left hand column) and 542 particles from the Cl treated sample. In this analysis height and width were determined by fitting a bounding rectangle to the particle outlines. The untreated particles are found to have a mean width of 5.06 ± 0.52 nm and an mean height of 4.93 ± 0.55 nm; while the treated particles are found to have an mean width of 4.58 ± 0.76 nm and an mean height of 4.57 ± 0.70 nm. All errors quoted correspond to one standard deviation. Histograms of the resulting data show that both sets of nanoparticles have a relatively narrow size distribution and that the majority of particles are close to spherical in shape having aspect ratios close to 1 (mean aspect ratios of 1.03 and 1.01 for the untreated and the Cl treated respectively).

References.

1. W. K. Bae, J. Joo, L. A. Padilha, J. Won, D. C. Lee, Q. Lin, W. Koh, H. Luo, V. I. Klimov, and J. M. Pietryga, *J. Am. Chem. Soc.* **134,** 20160–20168 (2012).
2. D. N. Dirin, S. Dreyfuss, M. I. Bodnarchuk, G. Nedelcu, P. Papagiorgis, G. Itskos, and M. V. Kovalenko*, J. Am. Chem. Soc.* **136,** 6550–6553 (2014).

3**.** W.W. Yu, L. Qu, W. Guo, X. Peng, *Chem. Mat.*, **15,** 2854-2860(2003).

4. S.M. Fairclough, E.J. Tyrell, D.M. Graham, P.J.B. Lunt, S.J.O. Hardman *et al, J. Phys. Chem. C*, **116,** 26898-26907 (2012).

5. A.G. Shard, J. Wang and S.J. Spencer, *Surf. Interface Anal.*, **41**, 541–548 (2009).

6. A.G. Shard, *J. Phys. Chem. C*, **116**, 16806-16813 (2012).

7. A. H. Ip, S. M. Thon, S. Hoogland, O. Voznyy, D. Zhitomirsky, R. Debnath, L. Levina, L. R. Rollny, G. H. Carey, A. Fischer, K. W. Kemp, I. J. Kramer, Z. Ning, A. J. Labelle, K. W. Chou, A. Amassian & E. H. Sargent *., Nat. Nano.* **7,** 577–582 (2012).
8. A.J. Nelson, S.P.Frigo and R.A. Rosenberg, *J. Appl. Phys.,* **75**, 1632-1637 (1994).
9. A.B. Christie, I. Sutherland, J.M. Walls, *Surface Science*, **135**, 225-242 (1983).
10. S. Beke, K. Sugioka, K. Midorikawa, A. Peter, L. Nanai and J. Bonse, *J Phys D,* **43**, 025401 (1-6), (2010).
11. K. Katsiev, A.H. Ip, A. Fischer, I. Tanabe, X. Zhang *et. al., Adv. Mater*., **26**, 937-942 (2014).
12. E. Ramos, B. M. Monroy, J. C. Alonso, L. E. Sansores, R. Salcedo and A. Martínez, *J. Phys. Chem. C.,* **116**, 3988-3994 (2012).
13. S. J.O. Hardman, D. M. Graham, S.K. Stubbs, B.F. Spencer, E.A. Seddon, H.-T. Fung, S. Gardonio, F. Sirotti, M.G. Silly, J. Akhtar, P.O'Brien, D. J. Binks and W.R. Flavell, *Phys. Chem. Chem. Phys.,* **13,** 20275-20283 (2011).
14. M. Sykora, A. Y. Koposov, J. A. McGuire, R. K. Schulze, O. Tretiak, J. M. Pietryga and V. I. Klimov, *ACS Nano*, **4**, 2021-2034 (2010).
